# A Computational Fluid Dynamic Investigation of Inhomogeneous Hydrogen Flame Acceleration and Transition to Detonation

**DOI:** 10.1007/s10494-018-9977-4

**Published:** 2018-09-01

**Authors:** Reza Khodadadi Azadboni, Ali Heidari, Jennifer X. Wen

**Affiliations:** 1 Fire, Explosion and Fluid Dynamics Research Team, School of Mechanical & Automotive Engineering, Kingston University London, London, SW15 3DW UK; 2 Warwick FIRE, School of Engineering, University of Warwick, Coventry, CV4 7AL UK

**Keywords:** Flame acceleration, Deflagration to detonation transition, VCEFoam, Mixtures with concentration gradients, Numerical modelling

## Abstract

Gas explosions in homogeneous reactive mixtures have been widely studied both experimentally and numerically. However, in practice and industrial applications, combustible mixtures are usually inhomogeneous and subject to vertical concentration gradients. Limited studies have been conducted in such context which resulted in limited understanding of the explosion characteristics in such situations. The present numerical investigation aims to study the dynamics of Deflagration to Detonation Transition (DDT) in inhomogeneous hydrogen/air mixtures and examine the effects of obstacle blockage ratio in DDT. VCEFoam, a reactive density-based solver recently assembled by the authors within the frame of OpenFOAM CFD toolbox has been used. VCEFoam uses the Harten–Lax–van Leer–Contact (HLLC) scheme fr the convective fluxes contribution and shock capturing. The solver has been verified by comparing its predictions with the analytical solutions of two classical test cases. For validation, the experimental data of Boeck et al. (1) is used. The experiments were conducted in a rectangular channel the three different blockage ratios and hydrogen concentrations. Comparison is presented between the predicted and measured flame tip velocities. The shaded contours of the predicted temperature and numerical Schlieren (magnitude of density gradient) will be analysed to examine the effects of the blockage ratio on flame acceleration and DDT.

## Introduction

Fire and explosion in combustible mixtures have been widely studied both experimentally and numerically. Most of the gas explosions studies inside tubes have been carried out for industrial safety applications with the aim to describe general mechanisms of flame acceleration (FA) and transition from deflagration to detonation (DDT) [2].

One of the primary hazards in hydrogen energy applications is the formation of flammable vapour clouds which can propagate kilometres away from the source with the possibility of ignition, resulting in fire and explosions [2]. Explosion studies in uniform reactive mixtures have been widely carried out both experimentally and numerically. However, in practice and industrial applications, combustible mixtures are usually non-uniform (inhomogeneous), and subject to both vertical and horizontal concentration gradients.

Thomas [3] has given a comprehensive discussion on various forms of DDT and differentiates the terminology between the macroscopic and microscopic DDT. The large-scale macroscopic DDT includes the process from accelerating deflagration followed by detonation propagation. The small-scale microscopic DDT governs the actual onset of detonation at the point where the combustion process changes from diffusion controlled to shock heating controlled [3]. In this work, the term DDT is used in the broader definition and includes both acceleration and onset of detonation. Thomas [3] also studied weak DDT, which was not onset by a strong reflected shock wave. He identified the importance of non-isotropic and non-equilibrium turbulence to accelerate a deflagration and some hot spots which generate transverse waves, which merged to strong pressure waves capable of forming the required shock interaction complex known as the detonation.

The combustible mixtures pose a risk especially when an ignition source is available or when the pressure and temperature exceed the self-ignition limits [2]. In the past, only a few studies considered the effect of mixture inhomogeneity on the DDT behaviour. Recently, Boeck et al. [1] studied FA and DDT in a channel with vertical hydrogen concentration gradients. They found that the hydrogen flame accelerated faster in the presence of concentration gradients as comparing with a homogenous mixture in the same configuration within the studied cases. DDT was observed to result from the interaction between the reflected shock waves and the deflagration front. The present study aims to provide an appropriate numerical method to provide the dynamics of Deflagration to Detonation Transition (DDT) in inhomogeneous hydrogen-air mixtures and examine the effects of obstacle blockage ratio in DDT phenomena.

### Methodology

A density-based solver within OpenFOAM CFD toolbox [4] is developed to model FA and DDT from rhoCentralFoam for density-based Navier-Stokes equation for combustion modelling. A detailed reaction mechanism [5] and the Monotone Integrated Large Eddy Simulation (MILES) [6] technique for turbulent modelling is adopted. In the MILES methods, the viscous dissipation scale will be replaced by a grid scale and the dissipation energy can be dissipated by a numerically imposed viscosity [7].

Convective terms are evaluated through an adaptation of the second order central-upwind numerical scheme of Kurganov et al., [8, 9]. The Harten–Lax–van Leer–Contact (HLLC) [10] scheme is used to evaluate the convective fluxes contribution and for accurate shock capturing. The solver and numerical schemes are initially verified by solving the Sod’s shock tube problem [11] and Supersonic Wedge problem [12].

## Governing Equations

The standard governing equations for solving the reactive inhomogeneous mixture in a Eulerian framework can be listed as below:

Mass conservation equation:

1 $$ \frac{\partial \rho} {\partial} +\nabla .(\rho U)= 0 $$

Conservation of momentum (neglecting body forces)

2 $$ \frac{\partial (\rho u)}{\partial t}+\nabla \cdot [u(\rho U)]+\nabla p+\nabla \cdot \tau = 0 $$

Conservation of total energy:

3 $$ \frac{\partial (\rho E)}{\partial t}+\nabla \cdot [u(\rho E)]+\nabla \cdot [up]+\nabla \cdot (\tau \cdot u)+\nabla \cdot j = 0 $$

Where in Eq. 1, *ρ* is the density, U the velocity, p the pressure and in the Eq. 2, *τ* is the viscous stress tensor which can be defined using Eq. 4 [13].

4 $$ \tau =-2\mu dev(D) $$

Where in Eq. 4, *μ* is the dynamic viscosity, D is the deformation gradient tensor which is defined as Eq. 5.

5 $$ D=\frac{1}{2}[\nabla u+(\nabla u)^{\tau} ] $$

In Eq. 3, j is the diffusive flux of heat, and E is the total energy density which can be defined as:

6 $$ E=e+\vert u\vert^{2}/2 $$

Where e, is the specific internal energy. Also, the ideal gas equation has applied.

7 $$ p=\rho RT $$

Where p is the pressure, *ρ* is the density, and T is the temperature of the mixture.

The specific gas constant R can be determined by the mean molar mass M of the mixture and the universal gas constant *R*:

8 $$ R=\frac{\Re} {M} $$

### Solution algorithms

Simulating discontinuities, such as shocks and contact surfaces in high-speed compressible flows requires numerical schemes that can capture these features while avoiding spurious oscillations. In some methods that are effective in producing accurate non-oscillatory solutions for capturing shock and discontinuities, the generation of numerical fluxes typically involves the Riemann solvers, characteristic decomposition and Jacobian evaluation, making them complicated and difficult to implement in a mesh of polyhedral cells that have an arbitrary number of faces [13]. However, an alternative approach exists which does not involve Riemann solvers and can also provide accurate non-oscillatory solutions using the so-called central schemes. The central schemes which are developed by Kurganov and Tadmor [8]; Kurganov et al. [9] and Greenshields et al. [13] are available in OpenFOAM as “rhoCentralFoam” [4, 8]. Khodadadi and Malekbala, [14] carried out shock capturing study using different OpenFOAM solvers to solve the sod’s problem [11] and concluded that rhoCentralFoam can provide reasonably accurate shock capturing. They also mentioned that the proposed density-based solution could generate some oscillations in the contact surface of shock. These oscillations may be linked to the numerical schemes.

On the basis of the “rhoCentralFoam” solver, a new solver called “VCEFoam” (VCEFoam is standing for; vapour cloud explosion in OpenFOAM) has been assembled [16]. It solves the compressible Navier–Stokes equations with either simple or detailed reaction mechanisms using the Monotone Integrated Large Eddy Simulation (MILES) approach. It uses a Godunov-type scheme implemented by Borm et al. [15], which uses the HLLC (Harten-Lax-van Leer-Contact) [10] scheme. For the time discrete schemes, which includes the dual time scheme and the physical time step, it uses the Runge–Kutta scheme [15]. In order to capture more small-scale features (such as Kelvin Helmholtz and Richtmyer Meshkov instabilities), the adaptive mesh refinement (AMR) has been facilitated. VCEFoam can simulate high Mach number reactive flows, with solving chemical reactions and HLLC schemes. To include the chemical reaction and species transport terms from the OpenFOAM’s combustion library, the energy equation needs to be changed from using the total energy to using the sensible enthalpy as in Eq. 7:

9 $$ \frac{\partial (\rho h_{s} )}{\partial t}+\nabla \cdot (\rho uh_{s} )-\frac{DP}{Dt}=\nabla \cdot \left[\alpha \nabla h_{s} +\sum\limits_{i = 1}^{n} {h_{i} J_{i}} \right]+\nabla \cdot (\tau \cdot u)=S_{h} $$

Where, u, P, hs, T, Sh, are the density, velocity, pressure, sensible enthalpy, temperature, and enthalpy source respectively. *α* is k/cp, k is the thermal conductivity and cp is the specific heat at constant pressure. The viscous stress tensor is defined in Eq. 4.

Species transport and diffusion coefficient are added, so the species conservation equation is:

10 $$ \frac{\partial (\rho Y_{i} )}{\partial t}+\nabla \cdot (\rho uY_{i} )=\nabla \cdot J_{i} +R_{i} $$

Where *J*_*i*_ is the diffusion flux of species i and is defined as Eq. 9;

11 $$ J_{i} =-\rho D_{i,m} \nabla Y_{i} $$

Where the binary diffusion coefficient, D_i,m_, for species i in the mixture can be derived by Wilke’s equation [17].

### Verification of numerical model

For verification of the developed numerical model, the Sod’s shock tube problem and supersonic wedge for oblique shock, have been simulated to verify the shock capturing capability and accuracy of the solver. the problem has been modelled.

#### Sod shock tube problem

**Fig. 1:**
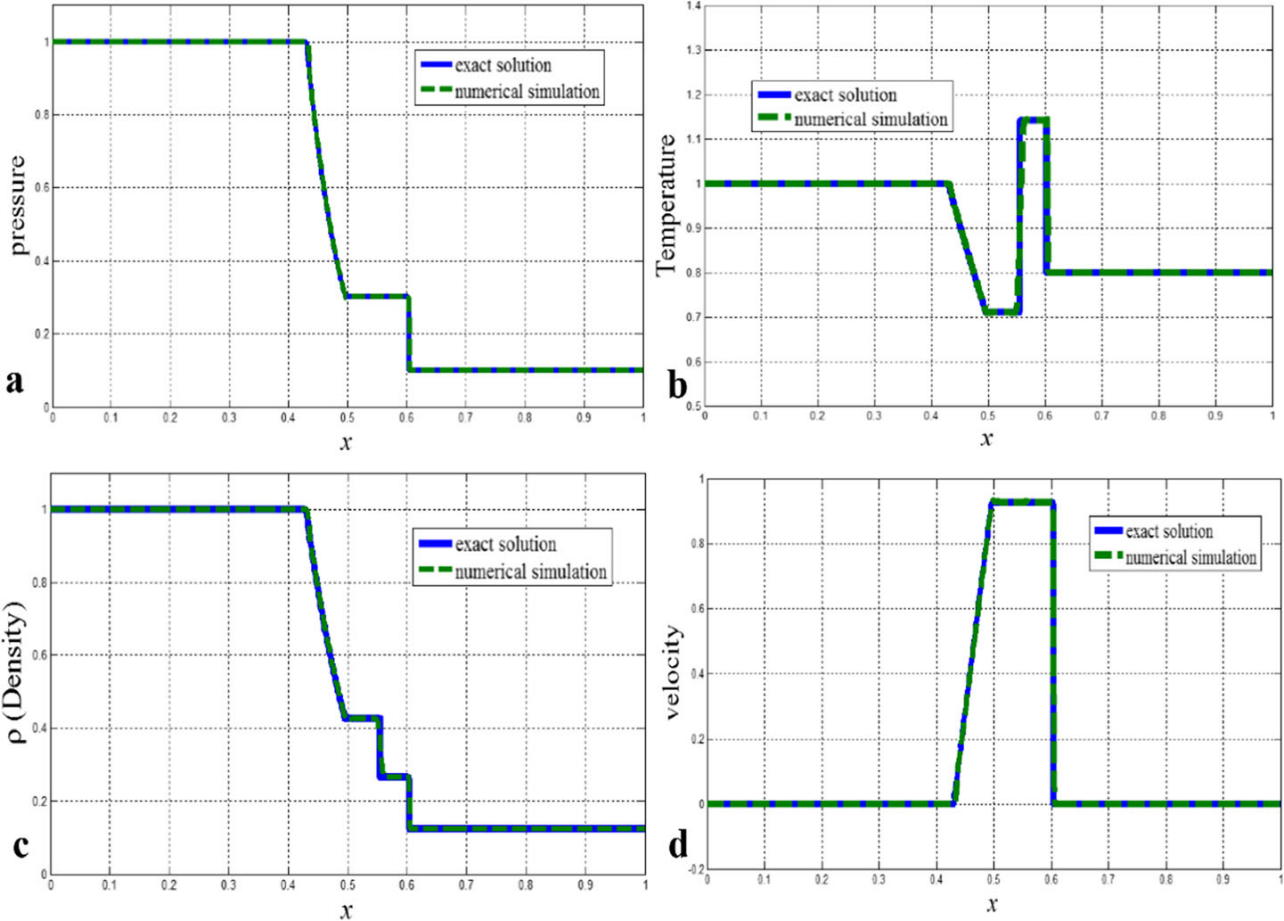
Density distribution in the shock tube with using 1000 cell; **a** pressure, **b** Temperature, **c** Density and **d** Velocity distributions

The Sod problem [11] was firstly chosen to validate the code regarding the ability of the code to capture discontinuities such as shock waves. The test case consists of a one-dimensional Riemann problem. As shown in Fig. 1, the predicted pressure, temperature, density, and velocity distributions are in excellent agreement with the analytical solutions. The predictions accurately capture the above mentioned important characteristics of the flow, i.e. the rarefaction wave, the contact discontinuity, and the shock discontinuity. No oscillations were found at the discontinuities. At the contact region, the predictions show steep gradients due to molecular diffusion which was captured by the code. The results in Fig. 1 shows a shock wave moving to the right and a rarefaction wave (expansion fan) moving to the left, the contact surface discontinuity separating the shock and rarefaction waves is moving to the right [11]. These results demonstrate that the newly assembled VCEFoam can provide accurate shock capturing.

#### The supersonic wedge problem

**Fig. 2:**
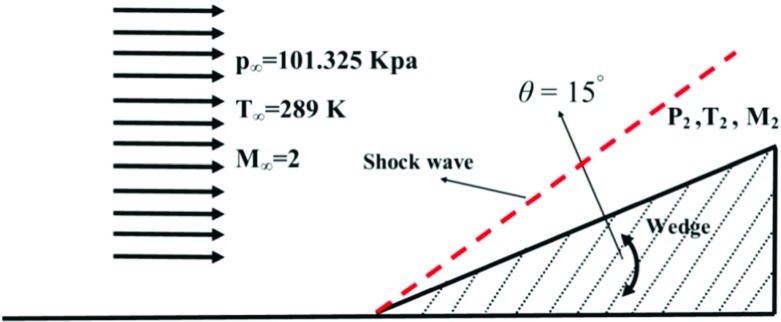
Computational domain of the supersonic wedge problem

The supersonic Wedge problem involving two-dimensional Oblique shock waves are often prevalent in the study of super- and hypersonic flows. It is hence crucial that a compressible flow solver can handle them well. Although one-dimensional Euler equations can represent the sod’s shock tube problem, the supersonic wedge problem requires two-dimensional equations. The computational domain is shown in Fig. 2. A horizontal stream with Ma= 2 meets a wedge whose axis of symmetry is parallel to the flow direction. The supersonic flow is then “turned into itself”, resulting in an oblique shock wave, after which the flow is again parallel and uniform. The wedge angle is chosen 15 degrees. The analytical solution of this problem is well known, and a MATLAB subroutine based on the theory in [12] has been coded to facilitate the comparison.

The initial conditions of the two-dimensional wedge problem, as well as the computational domain, is shown in Fig. 2. 50000 hexahedral cells have been considered for this simulation. In order to avoid reflection from the incoming waves, a wave transmissive boundary condition has been used for the outlet boundary condition. The upper boundary and the horizontal part of the lower boundary have been considered as being symmetric. For the rest of the regions a zeroGradient type, have been imposed.

**Fig. 3:**
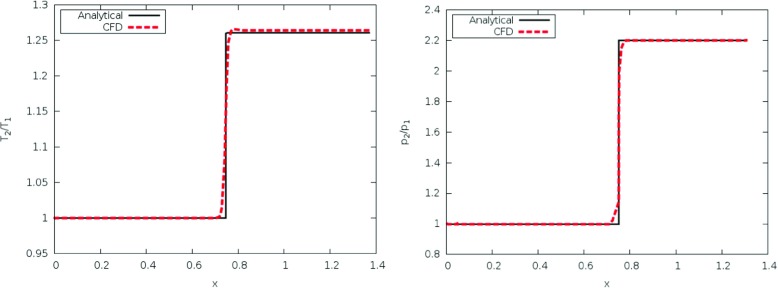
The predicted Temperature ratio distribution in the Wedge problem (right) and pressure ratio distribution in the Wedge problem (left)

**Fig. 4:**
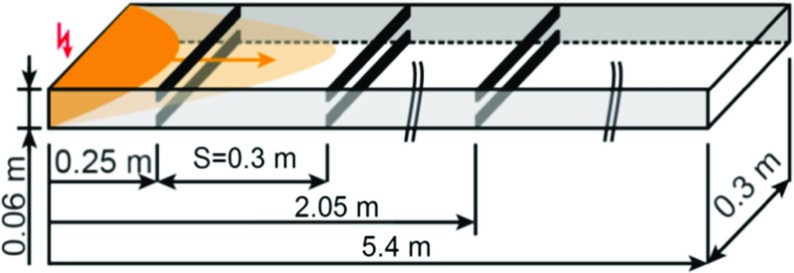
Schematic of the computational domain (Reproduced from Boeck et al. [1])

Figure 3 shows the predicted pressure and temperature ratios across the oblique shock. The predictions are in excellent agreement with the analytical solutions (Fig. 4).

## Results of the Validation Studies

For validation the test cases of Boeck et al. [1] were simulated. The experiments were conducted in a horizontal obstructed channel unobstructed channel (BR00) as well as 30% and 60% blockage ratios (BR30 and BR60). It was initially filled with an inhomogeneous hydrogen-air mixture, which was on average 40% hydrogen by volume.

### Numerical setup

The computational grid was set up to mimic the experimental configuration as shown in Fig. 1.

Three different sets of simulations have been carried out including an inhomogeneous hydrogen-air mixture with 40% concentration by volume (All the concentrations mentioned hereafter are by volume) in average with three different blockage ratio (BR) configuration, i.e. BR00, BR30 and BR60. In this study, the simulations have been carried out with adaptive mesh refinement, providing a minimum cell size of 10 μm, equivalent to approximately 30 grid points per half-reaction length. The simulations were conducted using a hydrogen/air reaction mechanism which contains 9 species and 21 detailed reactions [5].

#### Unobstructed channel with BR00

In this case, an unobstructed channel with an inhomogeneous hydrogen/air mixture with 40% averagre concentration, has been considered.

**Fig. 5:**
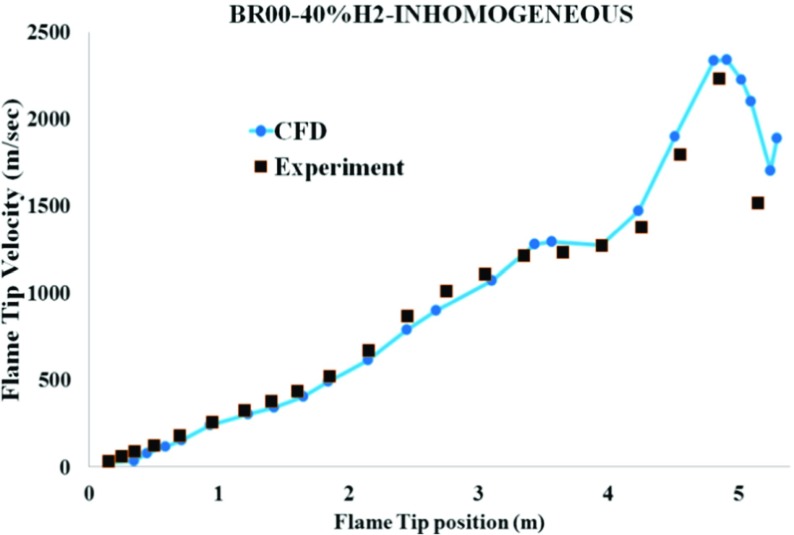
Comparison of the measured and predicted flame tip velocity for the inhomogeneous hydrogen/air mixture with 40 % averagre concentration for BR00

As shown in Fig. 5, the predicted flame tip velocities are in reasonably good agreement with the measurements. The flame tip velocity rises continuously in the channel.

**Fig. 6:**
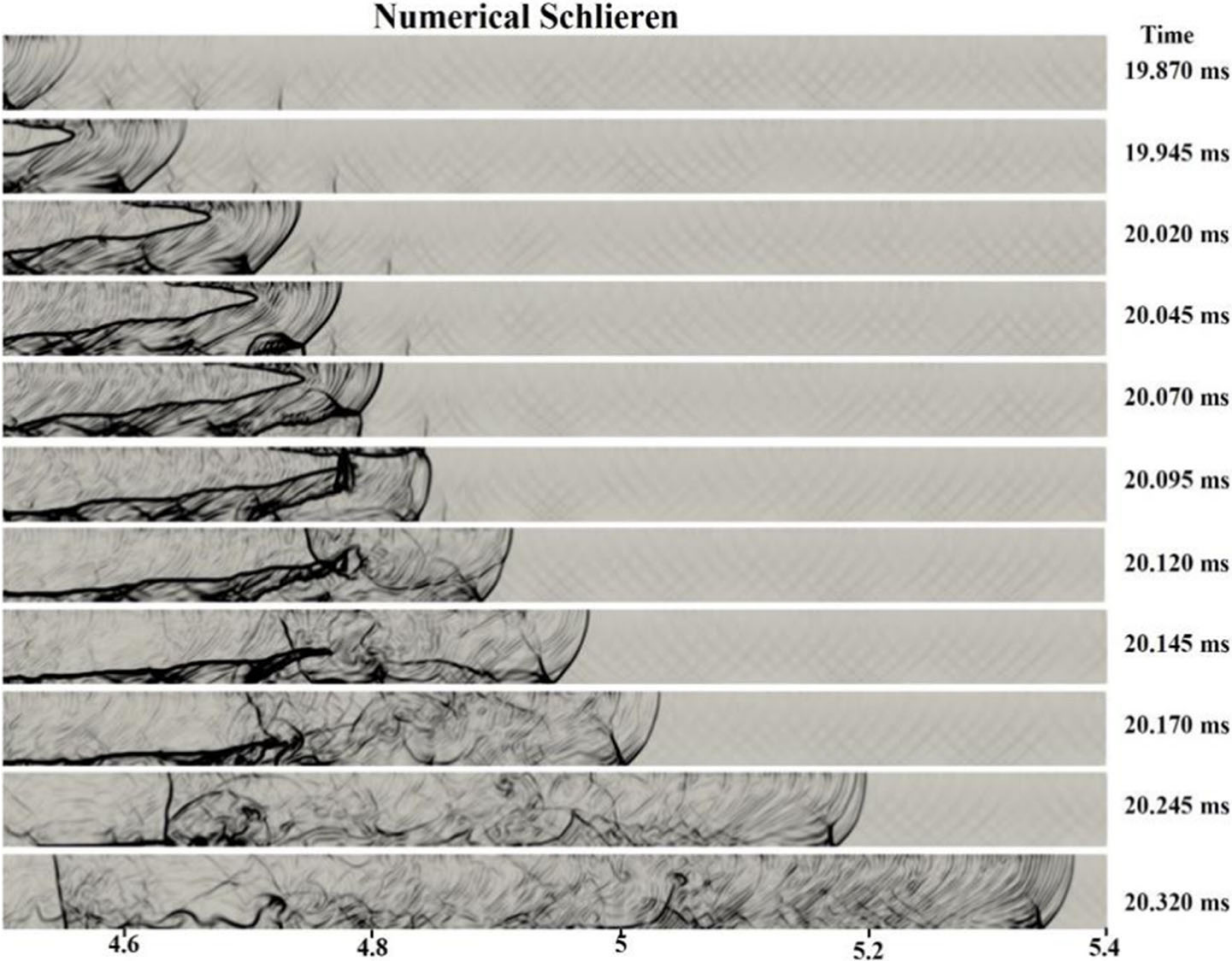
Numerical Schlieren for the inhomogeneous hydrogen/air mixture with 40% averagre concentration and BR00

**Fig. 7:**
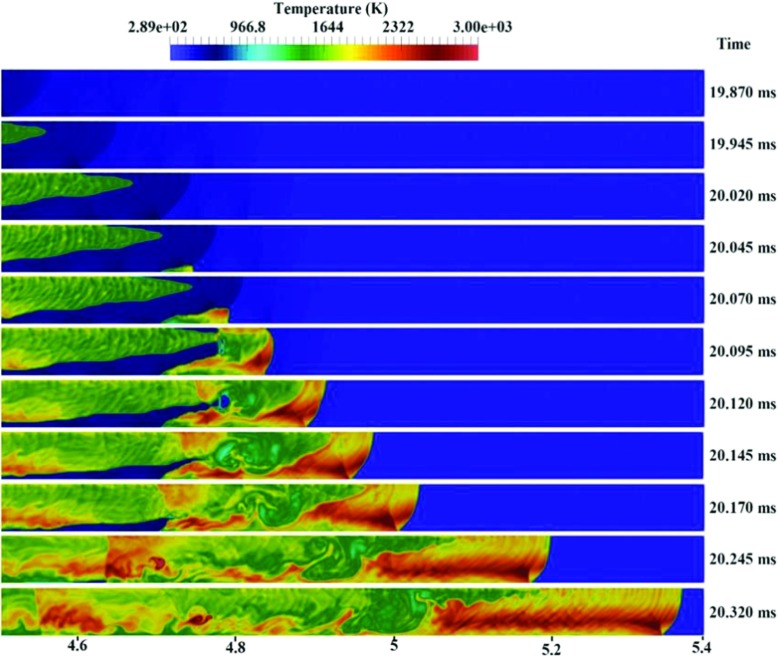
Temperature contour for the BR00 case

Based on Figs. 5 to 7, it can be found that in accordance with the experiments [1], the transition to detonation is initiated almost at the end of channel (x = 4.79m). It was triggered by shock reflection at the upstream faces from the channel wall, leading to local explosions in the leading shock wave. Figure 6 shows numerical schlieren images (magnitude of the density gradient) while Fig. 7 illustrates the temperature fields during DDT. It can be seen that the accelerated hydrogen flame is propagating with a leading shock ahead. Suddenly at 20.045ms, a weak local explosion occurs near the bottom wall where the mixture is lean. Then, at 20.095ms, this local explosion interacts with the flame front and DDT happens. The predicted pressure jumped over 20 bar in the results file (not shown here), which satisfies the CJ detonation pressure ratio condition. Moreover, Fig. 5 also shows that around this location (x = 4.8m), the flame tip velocity is at its maximum. At 20.120ms, the flow became supersonic, and the deflagration completely transited to detonation.

#### Obstructed channel with BR30

**Fig. 8:**
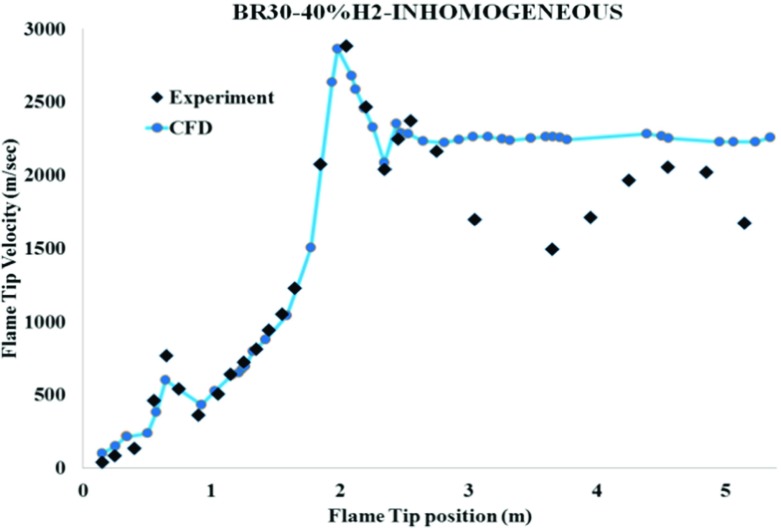
Comparison of the measured and predicted flame tip velocity for the inhomogeneous hydrogen/air mixture with 40 % averagre concentration and BR30

**Fig. 9:**
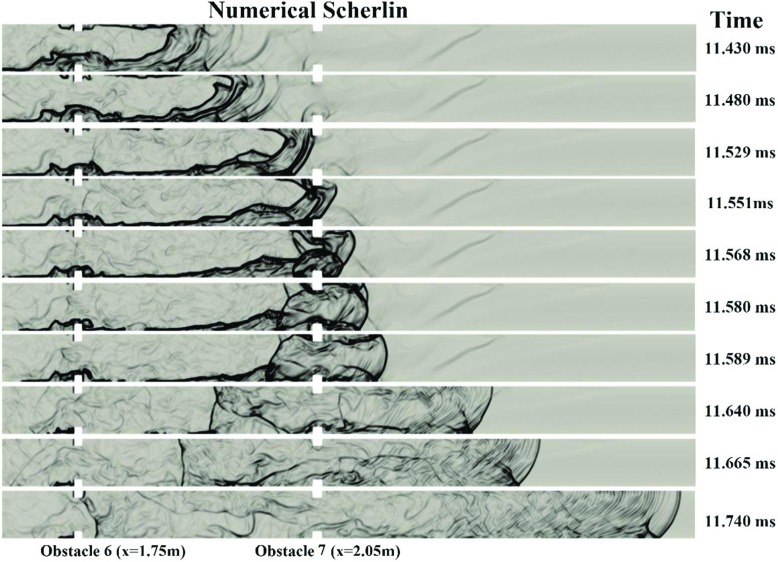
Numerical schlieren for the inhomogeneous hydrogen/air mixture with 40% averagre concentration and BR30

Figure 8 shows that the predicted flame tip velocities are in reasonably good agreement with the measurements of the 40% hydrogen concentration in a channel with 30% blockage ratio. In comparison with the unobstructed channel in Fig. 5, it can be seen that the flame has transited to detonation much earlier (in BR00, DDT occurred at x = 4.79m, however in BR30, the DDT took place at around x = 2.15m). Therefore, it can be concluded that obstruction has a significant impact on f FA and DDT. It is also see in Fig. 9 that the flame has transited to detonation.

#### Obstructed channel with BR60

**Fig. 10:**
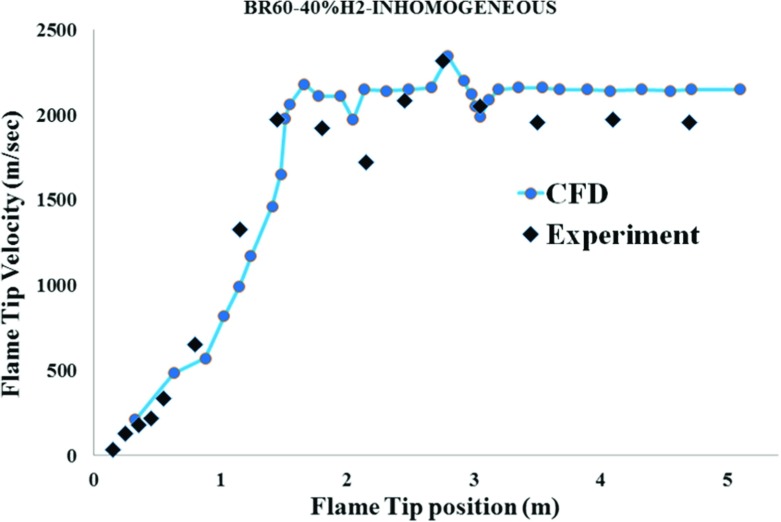
Comparison of the measured and predicted flame tip velocity for for the inhomogeneous hydrogen/air mixture with 40% averagre concentration and BR60

As shown in Fig. 10, the predicted flame tip velocities are in reasonably good agreement with the measurements. By comparing the results in Fig. 10 with that in Figs. 5 and 9, it can be seen that the maximum flame tip velocity occurred much earlier in the 60% BR case than the 30% BR and unobstructed cases. Also, the maximum flame speed in the BR30 case occurred sooner than the unobstructed case, indicating that within the tested range of between 0% to 60% BR, increasing the blockage ratio would lead to faster FA.

**Fig. 11:**
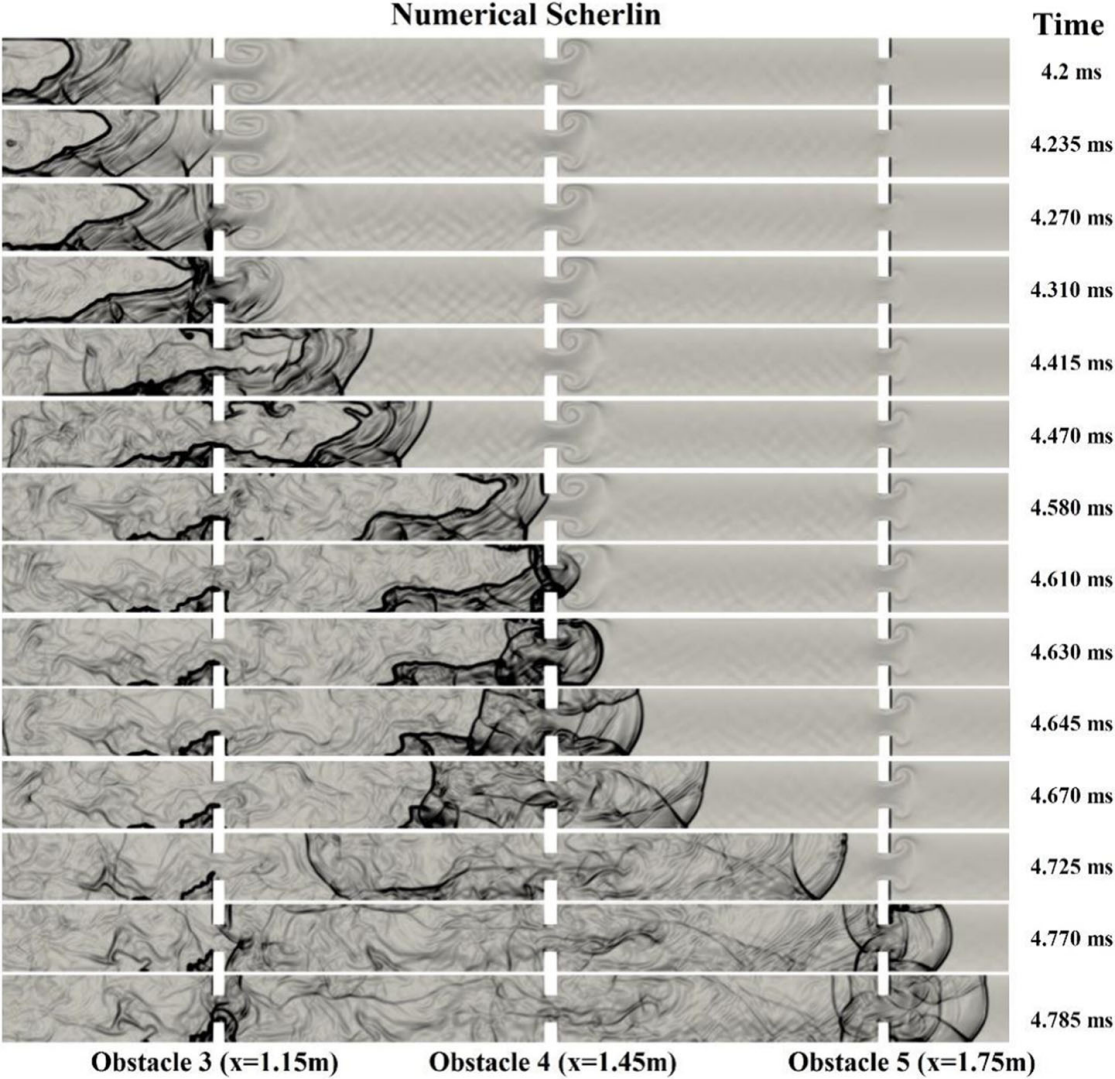
Numerical schlieren (magnitude of density gradient) contours for the inhomogeneous hydrogen/air mixture with 40% averagre concentration and BR60

Figure 11 shows the numerical schlieren. It can be seen that the onset of detonation occurred around obstacle 3, 4 and 5. At 4.2ms, a leading shock is moving downstream of the flame front. Then due to an interaction of the reflected shock from the third obstacle with the flame front, the first local explosion happened near the upper wall at 4.31ms. However, that explosion was not strong enough to trigger DDT and the flame was still decoupled with the shock wave. At 4.58ms, the second strong explosion happens due to the interaction of the reflected shock from obstacle 4 and the burned gas. At 4.61ms this explosion wave generated a third strong explosion in the middle of the channel. Subsequently, the DDT occurred at 4.62ms. At 4.645ms, two reflected shock waves generated in the downstream of obstacle 5 interacted in the middle of the channel, resulting in stronger leading shock waves. The process eventually reached steady state, and the shock wave propagated at nearly the C-J velocity.

Figure 11 also illustrated that in the BR60 channel, the transition of hydrogen flame to detonation happens earlier than the other cases with less blockage ratio. Therefore, it can be concluded that with the blockage ratio increasing from 0% to 60%, the speed of FA increase and the DDT occurs in less distance to the ignition point.

## Conclusions

The dynamics of FA and DDT in inhomogeneous mixtures have been studied using VCEFoam, a newly developed density-based solver within the frame of OpenFOAM CFD toolbox. To evaluate the convective fluxes contribution, Harten–Lax–van Leer–Contact (HLLC) scheme is used for accurate shock capturing. The solver has been firstly verified with the analytical solutions of the classical one-dimensional SOD’s shock tube problem and two-dimensional supersonic wedge case. For validation, the experimental data of Boeck et al. [1] is used. The experiments were conducted in a rectangular channel with three different blockage ratios. The predicted flame tip velocities were found to be in reasonably good agreement with the experimental measurements [1]; and the predicted FA and DDT behaviour are also in line with the experimental observations. The increase in the blockage ratio was found to increase the FA and reduce the running up distance to DDT. The results of the present work can be used in the context of safety to assess the potential risks of explosions in the energy industry. Also, in the future work, the effect of concentration gradients on DDT phenomena in a channel with and without obstructions, will be examined.

## Nomenclature

c_p_,: specific heat at constant pressure
*D*,: deformation gradient tensor
*E*,: total energy
e,: specific internal energy
h_s_,: sensible enthalpy
J,: diffusion flux
M,: mean molar mass
Ma,: Mach number
*P*,: pressure
R,: specific gas constant
S_h_,: enthalpy source
*t*,: time
T,: temperature
*U*,: velocity
Y,: species mass fraction
*α*,: thermal conductivity
*ρ*,: density
*τ*,: viscous stress tensor
*μ*,: dynamic viscosity
*𝜃*,: Wedge angel
ℜ,: universal gas constant.

## Subscripts

i,: species index
*∞*,: environmental properties
m,: mixture.
